# Breast Stimulation in Low-Risk Primigravidas at Term: Does It Aid in Spontaneous Onset of Labour and Vaginal Delivery? A Pilot Study

**DOI:** 10.1155/2014/695037

**Published:** 2014-11-27

**Authors:** Nilanchali Singh, Reva Tripathi, Yedla Manikya Mala, Niharika Yedla

**Affiliations:** Department of Obstetrics and Gynaecology, Maulana Azad Medical College and Associated Lok Nayak Hospital, Bahadur Shah Zafar Marg, Delhi Gate, New Delhi 110002, India

## Abstract

*Aims*. The aim of the study was to elicit the safety and efficacy of breast stimulation as an intervention to prevent postdatism and as an aid in spontaneous onset of labour. *Methods*. Primigravidas with cephalic presentation, without any high-risk factor, were recruited between 36 to 38 weeks of gestation. 200 patients were recruited and randomized into two groups (*n* = 100). Breast stimulation was advised to one group but not to the other group. Bishop's scoring was done at 38 weeks and repeated at 39 weeks of gestation. Maternal and fetal outcomes were compared in two groups. *Result*. Bishop's score changed from 3.12 (±1.01) to 3.9 (±1.08) in control group and from 3.02 (±0.82) to 6.08 (±1.29) in breast stimulation group after one week (*P* value < 0.0001). The period of gestation at delivery was 39.5 (±2.3) weeks in control group and 39.2 (±2.8) weeks in intervention group (*P* value: 0.044). There were increased chances of vaginal delivery in intervention group (*P* value: 0.046). Duration of labor, hyperstimulation, presence of meconium stained liquor, postpartum hemorrhage, and neonatal outcomes were similar in both groups. *Conclusion*. Breast stimulation in low-risk primigravidas helps in cervical ripening and increases chances of vaginal delivery.

## 1. Introduction

Spontaneous onset of labour has better maternal and fetal outcomes and more chances of vaginal delivery as compared to induced labour [1, 2]. To overcome the potential fetal and neonatal adverse effects of postdatism, induction of labor has been widely used. Many nonpharmacological methods have been used for cervical ripening since ages in different cultures and a recent survey suggests that substantial portion of women use these methods to induce labor [3]. Breast massage and nipple stimulation is one such method which has been studied by some authors, though the research is limited. Breast stimulation facilitates the release of oxytocin from posterior pituitary gland leading to cervical ripening. There is lack of evidence supporting breast stimulation as a viable method of labor induction. Few studies have demonstrated an abnormal fetal heart rate pattern after breast stimulation in high risk pregnancies [4]. Primigravidas usually have less favourable cervical findings as compared to multigravidas and are more likely to need interventions for cervical ripening. Hence we chose Low-risk primigravidas for our study. Breast stimulation is not as rapid as pharmacological methods in inducing labor and therefore, correct timing for start of this intervention is important. Cochrane reviews in 2012 suggest that delivery beyond 41 weeks of gestation is associated with more perinatal risks [5]. Breast massage is culturally more acceptable in Indian women. We have been advising breast stimulation to Low-risk patients after 38 weeks of gestation at our centre since decades and had observed favourable outcome. The efficacy and safety of breast stimulation are not proven yet and hence this study was conducted to portray the beneficial effect of breast stimulation and to standardize the technique of this manoeuver.

## 2. Methods

**Figure 1:**
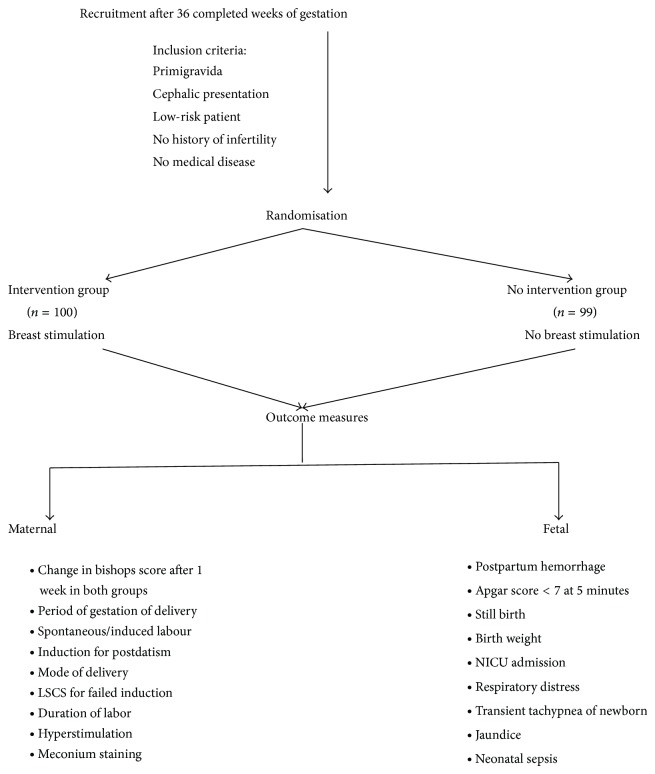
Study design.

The study was conducted at a tertiary care centre of North India over a period of six months in 2012. Ethical clearance was taken from the institutional ethical committee of Maulana Azad Medical College, New Delhi, where the study was conducted. 200 consecutive pregnant women attending antenatal clinic were recruited between 36 and 38 weeks of gestation after obtaining informed consent for inclusion in study. They were explained about the risks and benefits of the procedure including chances of early delivery. The inclusion criteria were primigravida with cephalic presentation, without any high risk factor. These high risk factors were gestational diabetes, hypertension, uncontrolled hypothyroidism, medical disease, or history of infertility. 200 patients were recruited and randomized into two groups of 100 each by computer generated sequence. A baseline cervical scoring using Bishop's score was done for all the patients at 38 weeks of gestation along with pelvic assessment. One group was assigned as intervention group and advised breast massage starting at 38 weeks of gestation. In an attempt to standardize the technique, the patients were shown a two-minute video of breast massage technique in which massage of whole breast from periphery towards centre including the nipple was performed using domestically available oil. The women were advised breast massage of each side for 15 to 20 minutes to be performed three times a day. They were advised to keep daily diary of manoeuvre and show on subsequent visit. The other group was assigned no intervention. The patients were advised to follow up weekly in antenatal clinic and report immediately to the obstetric casualty department in case of pain in abdomen, leaking or bleeding per vaginum, or in case of decreased fetal movements. At 39 weeks, repeat Bishop's score was done to see the change in cervical scores in both groups. All the vaginal examinations were done by the same author to exclude observer bias. The pelvic examination for Bishop's scoring was done by one of the authors in most patients. Those patients who had not done the manoeuvre as advised were excluded from the study. Those who have done a minimum of three massages for 10 minutes each were included in study. The maternal and fetal outcomes were observed in both groups. The medical personnel conducting delivery of these patients were blinded to the intervention. The study design is depicted in Figure 1.

Maternal outcomes were evaluated in the form of change in Bishops score after 1 week in both groups: period of gestation at delivery, onset of labor (Spontaneous/Induced), induction for postdatism, mode of delivery (Vaginal/LSCS), LSCS for failed induction, duration of labor, evidence of hyperstimulation, evidence of meconium stained liquor in labour, postpartum hemorrhage, and maternal satisfaction. Postdatism was defined as gestation beyond 41 weeks. Gestational age was calculated by first trimester ultrasound scan if the woman had one. If there was no first trimester scan available and she was sure of her last menstrual period with previous regular cycles, we used it for gestational age calculation. Duration of labour included both first (latent and active) and second stages of labour. Induction was done by intracervical dinoprostone gel (maximum three doses) followed by oxytocin infusion. Failed induction was defined as failure to progress to active phase of labour after 12 hours of amniotomy and/or oxytocin infusion. Our hospital follows the policy of active management of labour in all patients; hence, it was implemented in both of the study groups. Rigid inclusion criteria, strict diagnosis of labor, early amniotomy, frequent assessment of labor to ensure progress, and high-dose oxytocin for dystocia are the components of active management of labour. Occurrence of hyperstimulation (single contractions lasting 2 minutes or more or a contraction frequency of five or more in 10 minutes) and postpartum hemorrhage (loss of blood following childbirth resulting in hypovolemia or otherwise causing a woman to become symptomatic due to blood loss) were observed in both groups.

Neonatal outcomes were observed in the form of Apgar score < 7 at five minutes, requirement of neonatal intensive care unit (NICU) admission, neonatal morbidities like sepsis, asphyxia, jaundice, respiratory distress, and fetal or neonatal death.

The maternal and fetal outcomes in both of the groups were compared. SPSS Version 17 and Microsoft Excel 2007 have been used for statistical analyses. *χ* ^2^-test has been used for comparison of categorical variables and Student's *t*-test has been used for comparison of continuous variables. *P* values < 0.05 have been considered as significant. Odds ratios have been computed subsequently.

## 3. Results

There were 100 women in each group. One patient was lost to follow up in no intervention group and the rest of patients (*n* = 99) were analysed. The characteristics of patients in the intervention and no intervention groups are matched in Table 1. Maternal and fetal outcomes are mentioned in Tables 2 and 3, respectively. The significant results were change in Bishop's score after one week, period of gestation at delivery, and mode of delivery. The breast stimulation group had better results than no breast stimulation group in terms of these above mentioned parameters. The Bishop's score in control group changed from 3.12 (±1.01) to 3.9 (±1.08) after one week and from 3.02 (±0.82) to 6.08 (±1.29) in breast stimulation group and this finding was significant (*P* value < 0.0001). The period of gestation at delivery was 39.5 (±2.3) weeks in control group and 39.2 (±2.8) weeks in intervention group (*P* value: 0.044). There were increased chances of vaginal delivery in intervention group (*P* value: 0.046). Cesarean section for failed induction was 3.63 times more common in control group than in breast stimulation group, though the results were not statistically significant. Other indications of Caesarean section in both groups were cephalopelvic disproportion, fetal distress, and meconium stained liquor in early labour. The rate of induction for postdatism was also more common in control group. Postpartum hemorrhage was observed in six-percent cases in control group but none in intervention group. No significant difference was observed in fetal outcome in both groups.

Breast stimulation manoeuvre was acceptable to 92 patients in intervention group and they did not complain of any discomfort. Five patients had problems regarding privacy and space to perform this manoeuvre. Three patients had complaints of discomfort while performing breast stimulation.

## 4. Discussion

Breast stimulation is a nonpharmacological method which can be used for cervical ripening and increasing chances of onset of labour and hence avoidance of induction of labour for postdatism. Other potential benefits include prevention of protracted labour and postpartum hemorrhage [6, 7]. Many researchers have advocated nipple stimulation as a method of induction, but we believe that massage of whole breast including nipples is a more feasible and acceptable method among Indian women as it is culturally more acceptable. Also, in our prior experience, patients are more comfortable with breast stimulation, as compared to nipple stimulation, which is painful, at times. Induction of labour is associated with higher chances of Cesarean section [1]. Breast massage near term can reduce the need of labor induction with likelihood of patient going into spontaneous labor before 41 weeks gestation [8]. It can also lower the Cesarean section rates by decreasing inductions for postdatism and also increase chances of successful inductions. Cochrane systematic reviews, 2012, concluded that labour induction after 41 completed weeks is associated with fewer perinatal deaths as compared to continuation of pregnancy beyond [5]. Cochrane Systematic Reviews, 2010, analysed six trials and concluded that breast stimulation appears beneficial in Low-risk term pregnancies, but further research is required to evaluate its safety [7].

Bishop's score is a good indicator of preinduction of cervical status and a predictor of vaginal delivery; hence, it was used for studying the effect of intervention on cervical status in our study [9]. Our study results have shown significant change in Bishop's score after one week in breast stimulation group as compared to control group. There was a mean change of 3.60 ± 1.2 in Bishop's score after one week among the intervention group subjects compared to 0.78 ± 0.1 among the control group subjects in our study. In another study performed on 200 primigravidas evaluating the effect of unilateral breast stimulation, there was a mean change of 3.90 ± 2.39 points in cervical score among the study group subjects compared to 0.50 ± 0.67 among the control group subjects. When a cross-over trial involving 78 of the original 200 patients was performed, the study (ex-control) group had a mean change in cervical score of 3.84 ± 2.24 when compared with the control (ex-study) group, (1.43 ± 1.08) [10]. Our results were in conjunction with this study and it shows that breast stimulation has a definite role in cervical ripening.

Cesarean section was significantly less in breast stimulation group as compared to control group (8% versus 20.4%) which was statistically significant (*P* value: 0.046) though according to the Cochrane reviews, 2010, no significant difference was detected in the caesarean section rate (9% versus 10%, RR 0.90, and 95% CI 0.38 to 2.12) in such two groups [7]. Other advantage of breast stimulation is reduction in rates of postpartum haemorrhage. None of the patients had postpartum haemorrhage in intervention group as compared to 6.12% patients in control group in our study. A major reduction in the rate of postpartum haemorrhage was reported (0.7% versus 6%, RR 0.16, and 95% CI 0.03 to 0.87) in Cochrane Reviews [7].

Intrapartum hyperstimulation with or without nonreassuring fetal heart rate has been reported to be higher in breast stimulation group as compared to control group in some studies, whereas others have not reported any significant difference [4, 11, 12]. In our study one patient had hyperstimulation in breast stimulation group and none in the control group. Presence of meconium stained liquor was also not significantly different in both groups in our study as also observed in other studies [7]. We also found that fetal outcomes were similar in both groups. Similar results regarding fetal outcome was observed in other studies too [8, 11]. Our study did not report any adverse maternal or fetal effect in breast stimulation group and hence we found that breast stimulation is safe in Low-risk primigravida.

The usefulness of breast stimulation in high risk pregnancies including grand multiparas and previous Cesarean has been studied and found to be efficacious, but safety in these patients needs further evaluation [13]. We did not include high risk patients in our study group for uniformity of data. Cochrane reviews also suggest that until safety issues have been fully evaluated it should not be used in high-risk women. The reference to reductions in postpartum hemorrhage in the Cochrane Review includes studies in which nipple stimulation was performed intrapartum, which may not be applicable to this study, in which breast stimulation was performed well in advance of labor. Therefore, other potential benefits include prevention of protracted labour and postpartum hemorrhage, which need elucidation.

Maternal satisfaction rate was 92% and even those who were not satisfied had mild complaints. There was no discontinuation of intervention or withdrawal from study reported in this trial. Breast stimulation is also beneficial in terms of economic issues as compared to other pharmacological agents. It can be easily utilized in low resource settings to reduce further expenses as it minimizes chances of induction for postdatism (Odd's ratio: 3.16) and Caesarean section for failed induction (Odd's ratio: 3.63).

The strengths of the study are that there are very few randomized trials. Uniformity of data and standardization of maneuver have been done. The results are encouraging for use in low resource settings. The limitations are that sample size is less. There may be recruitment bias for subjects motivated to participate and an intervention bias whereby subjects randomized to the massage group may have been prompted to try other interventions, as there is no placebo arm. The assessment of cervical change was subjective. However, to minimize this observed bias, single author performed the vaginal examination. This study needs to be replicated in high risk groups in which Cesarean section rates are high. Further studies are indicated.

Breast stimulation in Low-risk primigravidas helps in cervical ripening and timely onset of labour without use of any pharmacological means. Even if the patients in breast stimulation group do not go into spontaneous labour, there are increased chances of successful induction for postdatism and vaginal delivery as the Bishop's score is likely to be higher. There were no maternal or fetal hazards associated with it as observed in this study. Hence, routine use of breast stimulation in Low-risk primigravidas may be a safe, efficient, and cost-effective intervention to avoid adverse perinatal effects associated with postdatism. Breast stimulation is economically beneficial as compared to other pharmacological agents; hence, it is useful in low resource settings.

## Figures and Tables

**Table 1:** Characteristics of patients in both groups.

Characteristics	No intervention (*N* = 99)	Intervention (*N* = 100)	*P* value
Age (in years)	27 ± 5.2	26 ± 6.1	0.74
Religion			
Hindu	52	49	
Muslim	45	51	Not significant
Others	2	0	
Educational status			
Illiterate	5	3	Not significant
Primary school	27	24	
High school	58	66	
Graduate	10	7	
BMI (in Kg/m^2^)	19.8 ± 2.2	20.8 ± 3.1	0.85

**Table 2:** Maternal outcome.

	No breast simulation (*N* = 99)	Breast simulation (*N* = 100)	*P* value	Odds Ratio
Mean Bishops at start/38 weeks	3.12 (±1.01)	3.02 (±0.82)	0.59	**—**
Mean bishops after 1 week	3.9 (±1.08)	6.08 (±1.29)	**<0.0001**	**—**
Change in Bishop score in 1 week	Increase of 25%	Increase of 101.3%	**0.0001**	**—**
Gestational age at delivery (in weeks)	39.5 (±2.3)	39.2 (±2.8)	**0.044**	**—**
Onset of labor				
Spontaneous	84 (85.7%)	92 (92%)	0.319	1.92
Induced	14 (14.3%)	8 (8%)		
Induction for postdatism	6 (6.06%)	2 (2%)	0.16	3.16
Mode of delivery				
Vaginal delivery	78 (79.6%)	92 (92%)	**0.046**	2.95
LSCS	20 (20.4%)	8 (8%)		
LSCS for failed induction	10 (10.1%)	3 (3%)	0.056	3.63
Mean duration of labour (in hours)	16.6 (±3.05)	17.28 (±3.63)	0.32	—
Hyperstimulation	0	1 (2%)	—	—
Meconium stained Liqour	3 (3.06%)	4 (4%)	0.78	0.75
Postpartum Hemorrhage	3 (6.12%)	0	**—**	**—**

Gestational age: period of gestation; LSCS: lower segment Cesarean section.

**Table 3:** Fetal outcome.

	No breast simulation (*N* = 99)	Breast simulation (*N* = 100)	*P* value	Odds ratio
Apgar score at 5 min (<7)	2 (2.05%)	3 (3%)	0.66	0.67
Mean birth weight (in grams)	2660 ± 381	2701 ± 360	0.58	—
Still birth	1 (1.02%)	1 (1%)	0.49	1.02
NICU^*^ admission	4 (4.1%)	6 (6%)	0.66	0.67
Respiratory distress	1 (1.02%)	1 (1%)	0.317	1.02
Jaundice	1 (1.02%)	1 (1%)	0.49	1.02
Transient Tachypnea of newborn	2 (2%)	1 (1.02%)	0.57	0.5

^*^NICU: Neonatal Intensive Care Unit.
